# Generation of Simian Rotavirus Reassortants with VP4- and VP7-Encoding Genome Segments from Human Strains Circulating in Africa Using Reverse Genetics

**DOI:** 10.3390/v12020201

**Published:** 2020-02-11

**Authors:** Alexander Falkenhagen, Corinna Patzina-Mehling, Ashish K. Gadicherla, Amy Strydom, Hester G. O’Neill, Reimar Johne

**Affiliations:** 1 Department of Biological Safety, German Federal Institute for Risk Assessment, 12277 Berlin, Germany; alexander.falkenhagen@bfr.bund.de (A.F.); Corinna.Patzina-Mehling@bfr.bund.de (C.P.-M.); Ashish.Gadicherla@bfr.bund.de (A.K.G.); 2 Department of Microbial, Biochemical and Food Biotechnology, University of the Free State, Bloemfontein 9301, South Africa; strydoma@ufs.ac.za (A.S.); OneillHG@ufs.ac.za (H.G.O.)

**Keywords:** rotavirus, reassortment, VP4, VP7, plasmid-based reverse genetics system, SA11, zoonosis, vaccine

## Abstract

Human rotavirus A (RVA) causes acute gastroenteritis in infants and young children. The broad use of two vaccines, which are based on RVA strains from Europe and North America, significantly reduced rotavirus disease burden worldwide. However, a lower vaccine effectiveness is recorded in some regions of the world, such as sub-Saharan Africa, where diverse RVA strains are circulating. Here, a plasmid-based reverse genetics system was used to generate simian RVA reassortants with VP4 and VP7 proteins derived from African human RVA strains not previously adapted to cell culture. We were able to rescue 1/3 VP4 mono-reassortants, 3/3 VP7 mono-reassortants, but no VP4/VP7 double reassortant. Electron microscopy showed typical triple-layered virus particles for the rescued reassortants. All reassortants stably replicated in MA-104 cells; however, the VP4 reassortant showed significantly slower growth compared to the simian RVA or the VP7 reassortants. The results indicate that, at least in cell culture, human VP7 has a high reassortment potential, while reassortment of human VP4 from unadapted human RVA strains with simian RVA seems to be limited. The characterized reassortants may be useful for future studies investigating replication and reassortment requirements of rotaviruses as well as for the development of next generation rotavirus vaccines.

## 1. Introduction

Rotavirus A (RVA) is the main causative agent of acute viral gastroenteritis in children under 5 years of age. Dehydration caused by severe diarrhea or vomiting can become life-threatening and it is estimated that rotavirus infections resulted in 128,500 deaths in 2016, of which 104,733 occurred in sub-Saharan Africa [1]. RVA is a non-enveloped virus with a double-stranded RNA (dsRNA) genome consisting of eleven segments encoding six structural viral proteins (VPs) and six non-structural proteins (NSPs) [2]. The VPs assemble into three concentric capsid layers. The middle and outer layer are formed by VP6 and VP7, respectively. VP4 forms spikes that are anchored in a cavity formed by VP6 and VP7 and protrude from the outer layer [3]. VP4 mediates entry into host cells and must undergo tryptic cleavage to acquire efficient infectivity for susceptible cells [4]. The cleavage generates an N-terminal, receptor-binding fragment called VP8* and a C-terminal fragment called VP5*. VP7 and VP4 represent the major rotavirus antigens that are capable of eliciting neutralizing antibody responses [5]. A binary typing system based on the nucleotide sequences of genome segments encoding the VP7 glycoprotein (G genotypes) and the protease-sensitive VP4 (P genotypes) is commonly used. A large variety of VP7 and VP4 has been described, allowing a classification into 36 G and 51 P genotypes, respectively [6]. Additionally, a classification system based on the nucleotide sequence of all eleven genome segments has been established, which indicates that the majority of RVAs worldwide have a Wa-like (Gx-P[x]-I1-R1-C1-M1-A1- N1-T1-E1-H1) or a DS-1-like (Gx-P[x]-I2-R2-C2-M2-A2-N2-T2-E2-H2) genotype constellation [7,8,9].

There are currently four approved vaccines available. Two of these vaccines (Rotarix and RotaTeq) have been used extensively globally over the past years. Rotarix is a live-attenuated vaccine derived from a human G1P[8] rotavirus isolate [10], while RotaTeq is a live-attenuated, pentavalent vaccine consisting of five human bovine reassortants (human G1, G2, G3, or G4 in a bovine background as well as human P[8] in a bovine background) [11]. The human parent G4 strain was originally isolated from a patient in France, but all other strains originate from the United States [12]. The effectiveness of both vaccines ranges from 85–98% in high-income countries in America, Asia and Europe, but was only modest in low-income countries in Africa and Asia (50–64%) [13]. Various reasons have been proposed for the reduced vaccine effectiveness in these regions including malnutrition, differences in gut microbiota, co-infections with other pathogens, or host genetic factors such as histo-blood group antigens (HBGAs) [14]. However, the vaccines could also have a lower efficacy against rotavirus strains circulating in Africa and Asia, which are considerably more diverse than those strains circulating in other regions and countries [15,16]. It has been shown that a reduction in G1P[8] strains occurred with an increase in uncommon strains in Australia and South Africa in the post-vaccination era [17,18]. The reduction in G1P[8] strains was specifically seen with Rotarix (G1P[8]), which might indicate different efficiencies of the vaccines against different genotypes. Nevertheless, more research is required to elucidate the reasons for the reduced vaccine efficiency in Africa and Asia as well as the characteristics of the strains circulating in these regions.

Adapting primary rotavirus strains to replicate in continuous cell lines is notoriously difficult and time consuming [19,20,21,22], which hinders the propagation and characterization of strains originating from Africa and Asia. For targeted generation of replicating rotaviruses, including specific recombinants and reassortants, several reverse genetics systems (RGS) have been developed [23,24,25]. Recently, fully plasmid-based systems for the generation of a simian RVA strain [26,27,28] and two human cell culture-adapted RVA strains [29,30] have been published. Using the simian RVA system, we have previously shown that VP4 from diverse animal rotavirus strains can be used to successfully generate viable reassortants [31].

Here, the generation of simian RVA reassortants with VP4- and VP7-encoding genome segments of three human RVA strains from Africa, which were not previously adapted to cell culture, was attempted. The generated reassortants were characterized according to their particle morphology and growth kinetics in cell culture. The reassortants may be useful for future studies investigating the requirements for reassortment and efficient replication of rotaviruses as well as for the targeted development of next generation vaccines with defined antigenicity.

## 2. Materials and Methods

### 2.1. Cell Lines

All cell culture reagents were obtained from Pan-Biotech GmbH (Aidenbach, Germany) unless indicated otherwise. Dulbecco’s Modified Eagle’s Medium (DMEM) and Minimal Essential Medium (MEM) were supplemented with 10% fetal calf serum (FCS), 1x nonessential amino acids, 2 mM L-glutamine, and 0.1 µg/mL gentamicin (hereafter referred to as complete DMEM and complete MEM). BSR T7/5 cells [25] were kindly provided by Karsten Tischer (Free University of Berlin, Germany) and maintained in complete DMEM containing 1 mg/mL G418 (Biochrom, Berlin, Germany). MA-104 cells were provided by the European Collection of Authenticated Cell Cultures (Salisbury, UK) and cultured in complete MEM. All cells were incubated at 37 °C and 5% CO_2_.

### 2.2. Plasmids

The plasmids encoding the 11 SA11-L2 genome segments as well as the three helper plasmids pCAG-D1R, pCAG-D12L, and pCAG-FAST-p10 were a kind gift from Takeshi Kobayashi [12] and were obtained from Addgene (Watertown, Massachusetts, USA). The human VP4- and VP7-encoding plasmids contained an expression cassette consisting of *Xba*I and *Pst*I restriction sites at the 5′end, a T7-RNA-polymerase (T7RNAP) promoter, the complete genome segment encoding VP4 or VP7, a hepatitis delta virus (HDV) ribozyme sequence, a T7RNAP terminator, and a *Hin*dIII as well as a *Not*I restriction site at the 3′ end. The sequences for the promoter, ribozyme, and terminator are identical to that of a plasmid encoding VP4 from avian RVA strain 02000V2G3 (GenBank: KT239165). The cassettes encoding VP4 from human RVA strain RVA/Human-wt/ZAF/GR10924/1999/G9P[6] (GR10924/99) (GenBank: FJ183356.1) or VP7 from the human RVA strains GR10924/99, RVA/Human-wt/MOZ/0060a/2012/G12P[8] (Moz60a), and RVA/Human-wt/MOZ/0308/2012/G2P[4] (Moz308) (GenBank: FJ183360.1, MG926763.1, and MG926730.1, respectively) were synthesized, cloned into pUCIDT-Amp, and sequence verified by Integrated DNA Technologies (IDT, Coralville, Iowa, USA). The expression cassettes for VP4 from human RVA strain Moz60a (GenBank: MG926761.1) and VP4 from human RVA strain Moz308 (GenBank: MG926728.1) were synthesized as gBlocks gene fragments (IDT). Adenosine overhangs were added to the gBlocks using Takara Ex Taq (Takara Bio Inc, Kusatsu, Japan) and the fragments were cloned into pCR4-TOPO using a TOPO TA cloning kit (Thermo Fisher, Waltham, MA, USA) according to the manufacturer’s instructions. The expression cassettes encoding VP4-Moz60a or VP4-Moz308 were then used to replace the *Xba*I to-*Hin*dIII or *Pst*I-to-*Not*I fragments of the plasmid encoding VP7-Moz308, respectively. All plasmids were purified using a plasmid midi kit (Qiagen, Hilden, Germany) prior to transfection.

### 2.3. Generation of SA11 and Reassortant Virus Using Reverse Genetics

SA11 and reassortant viruses were generated as described previously [31]. Briefly, BSR T7/5 cells were co-transfected with 11 plasmids encoding the individual rotavirus genome segments and three helper plasmids encoding two vaccinia virus capping enzyme subunits as well as a small membrane fusion protein. The transfected cells were co-cultured with MA-104 cells in the presence of trypsin before the cells were frozen and thawed once. Trypsin was added to 2 mL of freeze/thaw supernatants to a final concentration of 100 µg/mL and the mixture was incubated for 1 h at 37 °C.

### 2.4. Passaging of Reassortants

Confluent MA-104 cells grown in 6-well plates were washed twice with PBS, the mixture was added, and the cells were incubated for 1 h at 37 °C and 5% CO_2_. The cells were washed once with 3 mL MEM and 2 mL incomplete MEM (*w*/*o* serum) containing 10 µg/mL trypsin were added. The cells were incubated for 6–9 days and monitored for signs of cytopathic effects by microscopy before freeze/thaw supernatants were harvested and the procedure was repeated for passaging of the viruses.

### 2.5. RT-PCR and qRT-PCR

Viral RNA was extracted from freeze/thaw supernatants with the NUCLISENS easyMAG system (bioMérieux, Marcy-l’Étoile, France) and digested with RNase-free DNase (Roche, Basel, Switzerland) according to the manufacturers’ instructions. RT-PCRs were performed with the Qiagen OneStep RT-PCR Kit according to the manufacturer’s instructions. The primer sequences for strain-specific detection of VP4 and VP7 genes were as follows: VP4-GR10924.99-F, 5′-GTT-CGT-CAG-ACT-CCG-TCA-GG-3′; VP4-GR10924.99-R, 5′-TCC- ACG-TCA-GCT-TCC-ATC-AC-3′; VP7-GR10294.99-F, 5′-ACC-GAT-GTT-GTT-GAT-GGT-GTG-3′; VP7-GR10294.99-R, 5′-ACA-TCT-GAG-CCA-CCG-ACT-TG-3′; VP7-Moz60a-F, 5′-TGG-ATG-TAC- GAC-AAC-CGA-CG-3′; VP7-Moz60a-R, 5′-GCA-TCG-TTG-TTG-GAT-CTG-CT-3′; VP7-Moz308-F, 5′-GGG-AAC-GGA-CTG-TAC-GGT-AA-3′; VP7-Moz308-R, 5′-TGC-ATT-CGG-TCC-ACC-AAC- TT-3′; VP7-SA11-X1-F, 5′-TAT-TAT-CCG-ACT-GAG-GCT-GCG-3′; and VP7-SA11-X1-R, 5′-GCA- ACG-TCG-CGT-CAT-ATT-TCA-3′. The primers for the detection of VP4-SA11 and qRT-PCR primers and conditions for the detection of NSP3-SA11 have previously been described [31]. A dilution series of pT7-NSP3SA11 was used to generate a standard curve and determine genome copy equivalents (GCEs)/mL. The primers used for RT-PCR were synthesized by IDT. Primers and probe for qRT-PCR were synthesized by TIB Molbiol (Berlin, Germany).

### 2.6. Electron Microscopy

10 µL of the virus stocks were adsorbed onto Formvar/carbon-coated copper grids (Plano GmbH, Wetzler, Germany) for 60 s. Excess liquid was then blotted with a filter paper and the grids were stained with 2% uranylacetate for further 60 s. The grids were dried and then examined with a Jeol 1400 Plus (Jeol, Tokyo, Japan) transmission electron microscope (TEM) operating at 120 kV. Imaging was performed using an Olympus Veleta G2 digital camera (EMSIS, Münster, Germany). Particle size was measured using iTEM software provided by Olympus.

### 2.7. Endpoint Dilution Assays

Freeze/thaw supernatants were serially diluted and activated in MEM containing 10 µg/mL trypsin for 30 min at 37 °C. Confluent MA-104 cells grown in a 96-well plate were washed twice with PBS before addition of the activated virus. The cells were incubated with the activated virus for 1 h at 37 °C and 5% CO_2_. Following the infection period, incomplete MEM containing 1 µg/mL trypsin was added. The lower trypsin concentration in comparison to the trypsin concentration used for passaging the virus was chosen to reduce the detachment of uninfected cells in 96-well plates, which was more pronounced than in 6-well plates. Plates for the titration of VP7 reassortants were analyzed after 4 days. Plates for the titration of the VP4 reassortant were incubated for 8 days because of slower replication kinetics. The Spearman and Kärber algorithm [30] was used for the calculation of the 50% tissue culture infectious dose (TCID50).

### 2.8. RVA Replication Kinetics

Culture supernatants containing 2 × 10^6^ GCEs of the indicated strain were activated and used to infect MA-104 cells grown in a T-25 cell culture flask as described for the endpoint dilution assay. Following the infection period, the cells were washed once with MEM and 5 mL incomplete MEM containing 1 µg/mL trypsin were added. Supernatant samples (1 mL) were taken at the indicated time points and 1 mL incomplete MEM with fresh trypsin was added to the flask. The supernatants were analyzed by qRT-PCR and endpoint dilution assay. The whole experiment was performed three times under identical conditions but starting on different days (three independent experiments).

### 2.9. Genome Sequence Analyses

Sequence alignments and construction of phylogenetic trees were performed using the Clustal W method as implemented in the MegAlign module of the Lasergene software package (DNASTAR Inc., Madison, Wisconsin, USA). The full-length VP4- and VP7-encoding genome segments of the strains used in this study were compared with the corresponding segments of the Rotarix and RotaTeq vaccine strains (GenBank: GU565088.1, GU565077.1, GU565066.1 and GU565055.1 for RotaTeq P[5]; JN849113.1 for Rotarix P[8]; JN849114.1 for Rotarix G1; GU565057.1 for RotaTeq G1; GU565068.1 for RotaTeq G2; GU565079.1 for RotaTeq G3; GU565090.1 for RotaTeq G4; GU565046.1 for RotaTeq G6) [12]. The VP4-or VP7-encoding genome segment of chicken group D rotavirus strain 05V0049 (Genbank: NC_014513.1) was included in the analyses as an outlier group [32].

### 2.10. Statistics

The data are presented as mean ± standard deviation (SD). To determine statistical significance, a two-tailed unpaired *t* test was used. Results with a p value below 0.05, 0.01, or 0.001 were considered significant and marked with one, two, or three asterisks, respectively.

## 3. Results

### 3.1. Selection of Human RVA Strains from Sub-Saharan Africa

**Figure 1:**
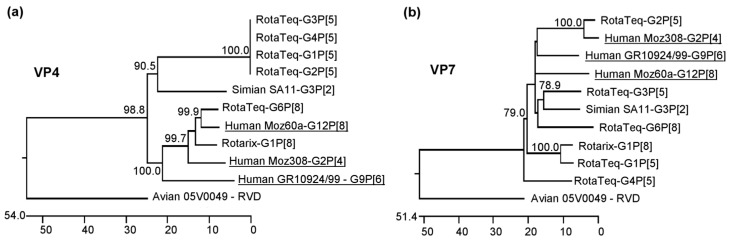
Phylogenetic relationship of the African strains used in this study with the vaccine strains present in Rotarix and RotaTeq and with the SA11 strain used in the reverse genetics experiments. (**a**) Phylogenetic tree based on the VP4-encoding genome segment. It is of note that the VP4-encoding segment belonging to the P[5] genotype in the RotaTeq vaccine strains is from a bovine RVA strain. (**b**) Phylogenetic tree based on the VP7-encoding genome segment. The VP7-encoding genome segment of the RotaTeq strain with the G6P[8] genotype is from a bovine RVA strain. Human African strains used in this study are underlined. The scale bar indicates the number of nucleotide changes per 100 nucleotides. The numbers in the phylogenetic trees indicate the bootstrap values from 1000 trials. The corresponding genome segments of rotavirus species D (RVD) were used as outliers.

VP4- and VP7-encoding genome segments of three RVA strains from sub-Saharan Africa were selected based on their genetic diversity and the availability of full-length genome sequences. Strain GR10924/99 [32,33] originated in South Africa, while strains Moz60a [34] and Moz308 [35] were identified in Mozambique. In all cases, the consensus genome sequences had been determined directly from fecal samples with no prior cell culture adaptation. GR10924/99, Moz60a, and Moz308 belong to the genotypes G9P[6], G12P[8], and G2P[4], respectively. GR10924/99 and Moz308 have a DS-1-like genotype constellation, while Moz60a has a Wa-like genotype constellation. The phylogenetic relationship of their VP4- and VP7-encoding genome segments with that of the strains present in the two major vaccines is shown in Figure 1. The VP4-encoding segment of Moz60a grouped with the corresponding segment of the G6P[8] strain from the RotaTeq vaccine, but none of the vaccine strains has a VP4-encoding genome segment belonging to the P[6] or P[4] genotype (Figure 1a). Based on the VP7-encoding genome segment, Moz308 clusters with the RotaTeq G2P[5] strain, but neither RotaTeq nor Rotarix contain a strain with a G9 or G12 genotype (Figure 1b). However, it is of note that different lineages exist within genotypes and that the VP4-encoding segment of Moz60a and the VP7-encoding segment of Moz308 belong to different lineages than the vaccine strains that they are grouping with in this analysis [36].

### 3.2. Sequence Analysis of Surface-Exposed Antigenic Regions of Human RVA Strains from sub-Saharan Africa

**Figure 2:**
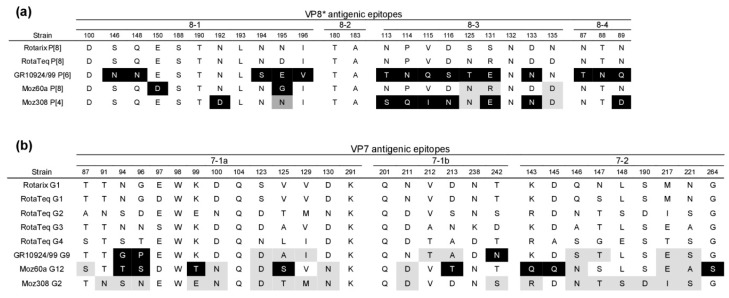
Comparison of the amino acid residues forming surface-exposed antigenic regions of the African strains used in this study and the vaccine strains present in Rotarix and RotaTeq. (**a**) VP8* antigenic regions. (**b**) VP7 antigenic regions. Black background: Residues different from Rotarix and RotaTeq. Light grey background: Residues different from Rotarix. Dark grey background: Residues different from RotaTeq.

VP8* is genetically diverse and contains four defined antigenic epitopes that are referred to as 8-1 to 8-4, while VP7 contains two epitopes that are referred to as 7-1 and 7-2, whereby the 7-1 epitope can be further divided into 7-1a and 7-1b [12,36,37]. A comparison of the amino acid residues that form the VP8* antigenic epitopes showed that GR10924/99 (G9P[6]) and Moz308 (G2P[4]) differed significantly from the vaccine strains present in Rotarix and RotaTeq (Figure 2a). Moz60a (G12P[8]) differed from either Rotarix or RotaTeq at only two residues, but was different from Rotarix in five positions (Figure 2a). An analysis of the residues constituting the VP7 antigenic epitopes showed that all three African strains differed largely from Rotarix, but differences to the strains present in RotaTeq were less pronounced (Figure 2b). Moz60a (G12P[8]) showed the greatest differences with 8/29 residues that were not present in Rotarix or RotaTeq (Figure 2b).

### 3.3. Generation of Simian RVA Reassortants with Human VP4 and VP7

**Figure 3:**
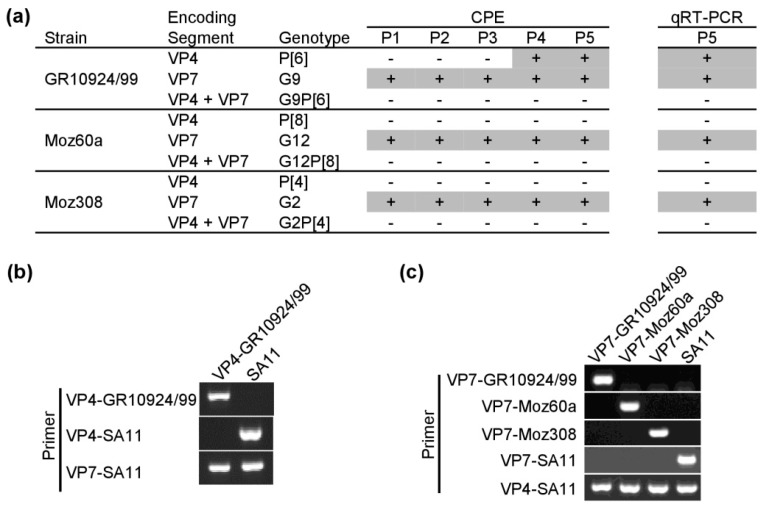
Generation of simian rotavirus A (RVA) reassortants with human VP4 and/or VP7. (**a**) Passaging of reassortants in MA-104 cells. Infected cells were analyzed by light microscopy for signs of CPE after 1–5 passages (P1–5). qRT-PCR analysis with primer pairs specific for the NSP3-encoding genome segment of SA11 was performed at P5. Plus signs indicate the presence of a clear CPE or a positive qRT-PCR signal. Results are representative of two independent experiments performed in duplicates. (**b**,**c**) RT-PCR analysis of viral RNA from the (**b**) VP4 reassortant and (**c**) VP7 reassortants using strain-specific primer pairs.

First, we tested whether the plasmid-based RGS can be used to generate simian SA11 reassortants with human VP4. To that end, the plasmid encoding simian VP4-SA11 was substituted with the plasmid encoding human VP4 from strain GR10924/99, Moz60a, or Moz308. Following the transfection of BSR-T7/5 cells and co-culture with MA-104 cells, freeze/thaw supernatants were passaged in MA-104 cells. However, using our previously published protocol [31], we were not able to rescue any VP4 reassortant. Only after using 20 times more freeze/thaw supernatant for passaging in MA-104 cells, we were able to rescue a simian SA11 reassortant with human VP4-GR01924/99. A clear cytopathic effect (CPE) was visible after four blind passages. The same CPE was observed upon further passaging of freeze/thaw supernatants and a positive qRT-PCR signal was detected, indicating that replication-competent virus was produced (Figure 3a). Therefore, all subsequent rescue attempts were made with the modified passaging protocol. However, even with the new protocol, we were unable to rescue reassortants with human VP4-Moz60a (G12P[8]) or VP4-Moz308 (G2P[4]).

Next, we attempted to rescue simian RVA reassortants with human VP7 using the modified protocol. In contrast to the results with VP4, we observed a strong CPE for all three VP7 reassortants in the first passage (Figure 3a). The reassortants stably replicated in MA-104 cells and samples tested positive in qRT-PCRs (Figure 3a). Lastly, we tried to rescue simian double reassortants with both human VP4 and VP7, but the attempts failed for all three strains (Figure 3a). Overall, we were able to generate four new simian SA11 reassortants, designated as VP4-GR10924/99, VP7-GR10924/99, VP7-Moz60a, and VP7-Moz308. The presence of the expected VP4-encoding genome segment (Figure 3b) or VP7-encoding segment (Figure 3c) was confirmed by RT-PCR using specific primer pairs.

### 3.4. Analysis of Reassortant Morphology by Electron Microscopy

**Figure 4:**
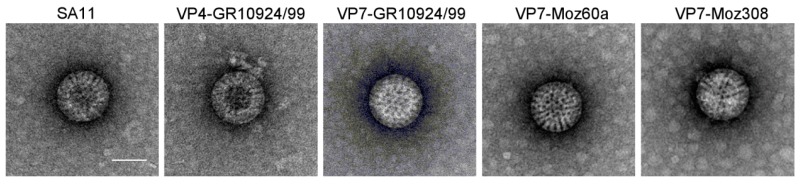
Morphology of the parent SA11 strain and the derived reassortants. Samples were stained with uranyl acetate and transmission electron microscopy was performed. Representative images of SA11 and the reassortants are shown. Scale bar = 50 nm.

To examine the virus particle morphology of the rescued reassortants, freeze/thaw supernatants from MA-104 cells infected with VP4-GR10924/99, VP7-GR10924/99, VP7-Moz60a, or VP7-Moz308 were analyzed by transmission electron microscopy. Typical triple-layered RVA-like particles were observed for the VP4 and VP7 reassortants and the morphology of the reassortants was indistinguishable from the morphology of the parent simian RVA strain SA11 (Figure 4). For all samples, the majority of the particles was intact and there were no apparent differences in the number of virus particles, although further quantitative analyses would be necessary to confirm this observation.

### 3.5. Replication Kinetics of Reassortants

**Figure 5:**
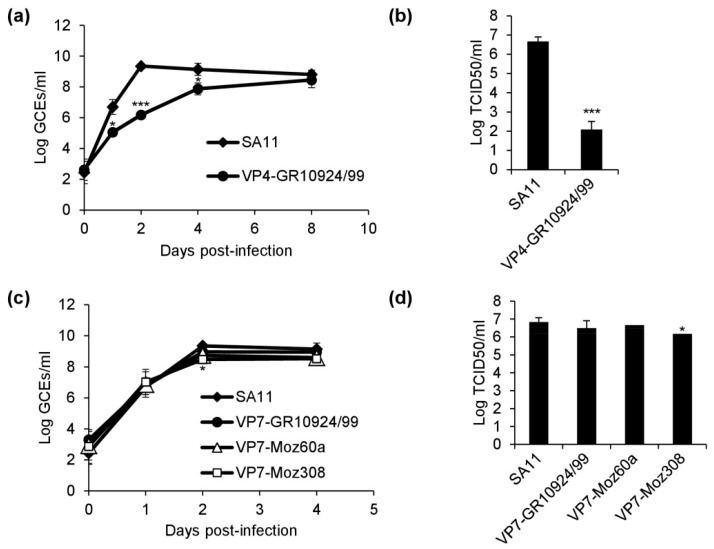
Replication kinetics of the generated reassortants. (**a**) Growth curve of VP4-GR10924/99 in comparison to SA11 based on genome copy equivalents (GCEs) determined by qRT-PCR. (**b**) Infectious titers determined by end-point dilution assay of VP4-GR10924/99 and SA11 at 8 days post-infection. (**c**) Growth curve of the VP7 reassortants in comparison to SA11 based on GCEs determined by qRT-PCR. (**d**) Infectious titers determined by end-point dilution assay of the VP7 reassortants and SA11 at 4 days post-infection. All data are means ± SD from three independent experiments. Statistical analysis was performed using Student’s *t*-test. * *p* < 0.05; *** *p* < 0.001.

In order to analyze the growth kinetics of the reassortants, we infected MA-104 cells at the same number of genome copy equivalents (GCEs) and analyzed supernatants from the infected cultures by qRT-PCR across multiple time points post-infection. In comparison to SA11, the VP4-GR10924/99 reassortant grew substantially slower (Figure 5a). The mean GCE number of VP4-GR10924/99 was significantly lower than that of SA11 at 1, 2, and 4 days post-infection, with a maximum difference of >3 logs at 2 days after infection. However, the mean GCE numbers between SA11 and VP4-GR10924/99 were similar at 8 days post-infection, the endpoint of the experiment. Samples from 8 days-post-infection were further analyzed by end-point dilution assay in order to determine the infectious titer (Figure 5b). Despite a similar number of GCEs at this time point, the infectious titer of VP4-GR10924/99 was 4 logs lower (*p* < 0.001). Assuming that the number of GCEs reflects the number of virus particles, this result might indicate that a high number of virus particles is accumulating in the cell culture supernatant, but that the majority of particles are not infectious.

In contrast, the replication kinetics for SA11, VP7-GR10924/99, VP7-Moz60a, and VP7-Moz308 were similar (Figure 5c). Only the mean GCE number of VP7-Moz308 was moderately reduced in comparison to SA11 at 2 days post-infection, but no significant difference was detected 4 days post-infection, the endpoint of the experiment. The infectious titers of samples harvested at 4 days post-infection were comparable as well, although a moderate reduction was detected for VP7-Moz308 in comparison to SA11 (Figure 5d).

## 4. Discussion

The recently established RGSs for RVA enable a targeted generation of viable reassortants as recently shown for SA11 containing VP4-encoding genome segments from diverse animal RVAs [31]. Of importance, the study described that one of the generated reassortants contained a VP4-encoding genome segments of a bat RVA, which was previously not adapted to cell culture [31]. Isolation of RVA in cell culture is generally difficult and usually requires passaging in primary cells [20]. Here, we have successfully generated reassortants with VP4 or VP7 from human RVA strains circulating in Africa. None of these strains had been adapted to cell culture before, but the generated reassortants efficiently grew in MA-104 cells. Therefore, this system can be a useful tool to study rotavirus proteins for which only sequence data is available so far in a context of infectious virus.

We were able to rescue all tested VP7 reassortants. Additionally, the growth kinetics of the rescued reassortants were similar. These results suggest a high compatibility of human VP7 with the simian backbone. VP7 reassortment between animal and human strains occurs naturally [38,39,40], but animal reassortants with human VP7 have also been generated in vitro by co-infection of cells with human and animal rotaviruses [41,42]. However, reassortants with VP7 from animal strains had to be repeatedly depleted using antisera or antibodies in order to select reassortants with human VP7 [41,42]. Using the plasmid-based RGS, we were able to rescue the VP7 reassortants after a single passage in MA-104 cells, highlighting the usefulness of the system to quickly generate reassortants. In contrast, rescue of reassortants with human VP4 was more difficult. We were only able to rescue one reassortant with VP4 from strain GR10924/99 (genotype P[6]). VP4-encoding segments belonging to genotype P[6] have been reported on both Wa-like and DS-1-like backbones and in combination with various G types in Africa [43], which could suggest a natural tendency for reassortment.

Early reassortment studies based on coinfection with simian rotaviruses and human rotaviruses were also unable to generate reassortants with human VP4 [41,44]. Similarly, co-infection with a bovine strain and unadapted human strains did not yield any reassortants with human VP4 [45]. Using a plasmid-based reverse genetics system, Kawagishi et al. were very recently able to rescue SA11 with VP4 from human strain Odelia (G4P[8]) [30]. This reassortant replicated very poorly in cell culture and grew to significantly lower titers than the parent human or simian strain. Similarly, our results showed that the VP4 reassortant replicated very poorly in cell culture in comparison to the simian strain. Comparing the replication also to the parent human strain would be ideal, but since we only had sequence data available for the strains used in this study, trials of adaptation to cell culture were not possible.

It is currently unclear why reassortment is restricted, but it will be crucial to determine the factors that govern successful reassortment. Gorell et al. [46] performed multiple rounds of negative selection with anti-human VP4 antibodies after co-infection with simian SA11 and human Wa (G1P[8]), but were not able to generate a reassortant with human VP4-Wa in an otherwise SA11 background [46]. However, the authors could rescue a multi-reassortant SA11 with VP2-Wa, VP4-Wa, and VP6-Wa. We have rescued the VP4 and VP7 mono-reassortants for strain GR10924/99 (G9P[6]), but were unable to rescue the corresponding double reassortant. Assuming that VP4 and VP7 of the same strain are compatible, this could also indicate that simian VP6 is a limiting factor, since VP6 and VP7 interact to form a pocket for VP4 [3].

We were unable to rescue reassortants with VP4 from Moz60a or Moz308 (genotype P[4] or P[8], respectively). VP4 contains the VP8 * domain, which mediates binding of cellular receptor molecules. HBGAs have been suggested as receptors for human RVAs and human RVA strains belonging to genotype P[6] were shown to bind to different HBGAs than strains belonging to the genotypes P[4] and P[8] [47,48]. However, the role of HBGAs in cell culture experiments remains controversial and seems to be dependent on the strain and the cell line used [49,50,51]. Entry especially in non-human cell lines such as MA-104 cells seems to be independent of HBGAs [49]. Nevertheless, the P[4] and P[8] strain could have different receptor requirements than the P[6] strain, which may be one reason for the different rescue success of the VP4 reassortants. Using a fully human plasmid-based RGS, Komoto et al. were only able to rescue virus after utilizing roller-tube cultures, which improved infection presumably by increasing attachment to cells [29]. Alternatively, using other cell lines, such as HT-29, Caco, ST-1 or HEK cells, which are susceptible to a broader range of human RVAs may improve rescue attempts in the future.

Neither the Rotarix nor the RotaTeq vaccine contain a strain with VP4 belonging to the P[6] genotype (GR10924/99) or VP7 belonging to the G9 (GR10924/99) or G12 (Moz60a) genotypes. Strains with the P[6] genotype are more prevalent in developing countries and are especially common in Africa [15]. For example, in Zambia during a surveillance period from 2008 to 2015, each year 24–66% of the typed RVA strains belonged to the P[6] genotype [52]. RVAs belonging to the G9 or G12 genotypes are also widespread in many regions in Africa [18,53]. For instance, the prevalence of G9 strains was 34% in Zimbabwe before vaccine introduction (2008–2013) and 35% after vaccine introduction (2015–2016) [54]. Therefore, the VP4 and VP7 reassortants generated here may be useful for the generation of vaccines based on strains circulating in Africa. Recently, two additional rotavirus vaccines (Rotavac and Rotasiil) have been developed in India [55,56]. Rotavac is based on strain 116E, which is a naturally-occurring G9P[11] human reassortant with bovine VP4 and was originally isolated from neonates with asymptomatic infection in India [57]. This vaccine strain belongs to the G9 genotype, but there is no human VP4 component. Rotasiil is a pentavalent vaccine similar to RotaTeq and consists of bovine reassortants with human G1-4 and human G9 [58]. However, none of the novel vaccine strains contains human VP4. As immune responses to VP4 can be important for protection from infection [59], homologous VP4 antigens may be preferable in vaccines.

In conclusion, we have shown that the plasmid-based RGS can be used to generate viable reassortants with VP4- and VP7-encoding genome segments from human field strains for which only consensus sequence data were available. Some of the VP4 reassortants could not be generated, indicating present limitations of the system, which should be investigated in more detail in future. The generated VP4 reassortant replicated significantly slower than the parent simian virus, emphasizing the importance of VP4 during replication in cell lines. Future studies should investigate the genetic stability of the strains in more detail by sequencing the whole genome of the reassortants before and after a large number of sequential passages in MA104 cells. As VP4 and VP7 are the major rotavirus antigenic determinants that elicit neutralizing antibody responses, further experiments should examine the antigenicity of the generated reassortants and compare it with that of the currently available vaccines. The VP4- and VP7-encoding genome segments chosen in our study originated from sub-Saharan Africa and may therefore represent well suited antigens in vaccines for this region. Ideally, a panel of reassortants representing common and uncommon genotypes present in Africa could be generated in the future. In addition, the characterized reassortants may generally be useful for future studies investigating replication and reassortment requirements of rotaviruses.

## References

[1] Troeger C., Khalil I.A., Rao P.C., Cao S., Blacker B.F., Ahmed T., Armah G., Bines J.E., Brewer T.G., Colombara D.V. (2018). Rotavirus Vaccination and the Global Burden of Rotavirus Diarrhea Among Children Younger Than 5 Years. JAMA Pediatr..

[2] Crawford S.E., Ramani S., Tate J.E., Parashar U.D., Svensson L., Hagbom M., Franco M.A., Greenberg H.B., O’Ryan M., Kang G. (2017). Rotavirus infection. Nat. Rev. Dis. Primers.

[3] Settembre E.C., Chen J.Z., Dormitzer P.R., Grigorieff N., Harrison S.C. (2011). Atomic model of an infectious rotavirus particle. EMBO J..

[4] Estes M.K., Graham D.Y., Mason B.B. (1981). Proteolytic enhancement of rotavirus infectivity: Molecular mechanisms. J. Virol..

[5] Desselberger U., Huppertz H.I. (2011). Immune responses to rotavirus infection and vaccination and associated correlates of protection. J. Infect. Dis..

[6] Rotavirus Classification Working Group. https://rega.kuleuven.be/cev/viralmetagenomics/virus-classification/rcwg.

[7] Matthijnssens J., Ciarlet M., Heiman E., Arijs I., Delbeke T., McDonald S.M., Palombo E.A., Iturriza-Gomara M., Maes P., Patton J.T. (2008). Full genome-based classification of rotaviruses reveals a common origin between human Wa-Like and porcine rotavirus strains and human DS-1-like and bovine rotavirus strains. J. Virol..

[8] Matthijnssens J., Mino S., Papp H., Potgieter C., Novo L., Heylen E., Zeller M., Garaicoechea L., Badaracco A., Lengyel G. (2012). Complete molecular genome analyses of equine rotavirus A strains from different continents reveal several novel genotypes and a largely conserved genotype constellation. J. Gen. Virol..

[9] Matthijnssens J., Ciarlet M., McDonald S.M., Attoui H., Banyai K., Brister J.R., Buesa J., Esona M.D., Estes M.K., Gentsch J.R. (2011). Uniformity of rotavirus strain nomenclature proposed by the Rotavirus Classification Working Group (RCWG). Arch. Virol..

[10] Ward R.L., Bernstein D.I. (2009). Rotarix: A rotavirus vaccine for the world. Clin. Infect. Dis..

[11] Heaton P.M., Goveia M.G., Miller J.M., Offit P., Clark H.F. (2005). Development of a pentavalent rotavirus vaccine against prevalent serotypes of rotavirus gastroenteritis. J. Infect. Dis..

[12] Zeller M., Patton J.T., Heylen E., de Coster S., Ciarlet M., van Ranst M., Matthijnssens J. (2012). Genetic analyses reveal differences in the VP7 and VP4 antigenic epitopes between human rotaviruses circulating in Belgium and rotaviruses in Rotarix and RotaTeq. J. Clin. Microbiol..

[13] Jonesteller C.L., Burnett E., Yen C., Tate J.E., Parashar U.D. (2017). Effectiveness of Rotavirus Vaccination: A Systematic Review of the First Decade of Global Postlicensure Data, 2006–2016. Clin. Infect. Dis..

[14] Desselberger U. (2017). Differences of Rotavirus Vaccine Effectiveness by Country: Likely Causes and Contributing Factors. Pathogens.

[15] Todd S., Page N.A., Duncan Steele A., Peenze I., Cunliffe N.A. (2010). Rotavirus strain types circulating in Africa: Review of studies published during 1997–2006. J. Infect. Dis..

[16] Patton J.T. (2012). Rotavirus diversity and evolution in the post-vaccine world. Discov. Med..

[17] Roczo-Farkas S., Kirkwood C.D., Cowley D., Barnes G.L., Bishop R.F., Bogdanovic-Sakran N., Boniface K., Donato C.M., Bines J.E. (2018). The Impact of Rotavirus Vaccines on Genotype Diversity: A Comprehensive Analysis of 2 Decades of Australian Surveillance Data. J. Infect. Dis..

[18] Page N.A., Seheri L.M., Groome M.J., Moyes J., Walaza S., Mphahlele J., Kahn K., Kapongo C.N., Zar H.J., Tempia S. (2018). Temporal association of rotavirus vaccination and genotype circulation in South Africa: Observations from 2002 to 2014. Vaccine.

[19] Arnold M., Patton J.T., McDonald S.M. (2009). Culturing, storage, and quantification of rotaviruses. Curr. Protoc. Microbiol..

[20] Ward R.L., Knowlton D.R., Pierce M.J. (1984). Efficiency of human rotavirus propagation in cell culture. J. Clin. Microbiol..

[21] dos Santos A.C.S., Benati F.J., Lauretti F., Linhares R.E.C., Nozawa C. (2014). Biological, Molecular and Immunocytochemical Characterization of Cell Culture Adapted Human Rotavirus Strains Detected in the City of Ponta Grossa, Parana, Brazil. Virus Rev. Res..

[22] Otto P.H., Reetz J., Eichhorn W., Herbst W., Elschner M.C. (2015). Isolation and propagation of the animal rotaviruses in MA-104 cells--30 years of practical experience. J. Virol. Methods.

[23] Johne R., Reetz J., Kaufer B.B., Trojnar E. (2016). Generation of an Avian-Mammalian Rotavirus Reassortant by Using a Helper Virus-Dependent Reverse Genetics System. J. Virol..

[24] Komoto S., Sasaki J., Taniguchi K. (2006). Reverse genetics system for introduction of site-specific mutations into the double-stranded RNA genome of infectious rotavirus. Proc. Natl. Acad. Sci. USA.

[25] Trask S.D., Taraporewala Z.F., Boehme K.W., Dermody T.S., Patton J.T. (2010). Dual selection mechanisms drive efficient single-gene reverse genetics for rotavirus. Proc. Natl. Acad. Sci. USA.

[26] Kanai Y., Komoto S., Kawagishi T., Nouda R., Nagasawa N., Onishi M., Matsuura Y., Taniguchi K., Kobayashi T. (2017). Entirely plasmid-based reverse genetics system for rotaviruses. Proc. Natl. Acad. Sci. USA.

[27] Komoto S., Fukuda S., Ide T., Ito N., Sugiyama M., Yoshikawa T., Murata T., Taniguchi K. (2018). Generation of Recombinant Rotaviruses Expressing Fluorescent Proteins by Using an Optimized Reverse Genetics System. J. Virol..

[28] Kanai Y., Kawagishi T., Nouda R., Onishi M., Pannacha P., Nurdin J.A., Nomura K., Matsuura Y., Kobayashi T. (2019). Development of Stable Rotavirus Reporter Expression Systems. J. Virol..

[29] Komoto S., Fukuda S., Kugita M., Hatazawa R., Koyama C., Katayama K., Murata T., Taniguchi K. (2019). Generation of Infectious Recombinant Human Rotaviruses from Just 11 Cloned cDNAs Encoding the Rotavirus Genome. J. Virol..

[30] Kawagishi T., Nurdin J.A., Onishi M., Nouda R., Kanai Y., Tajima T., Ushijima H., Kobayashi T. (2020). Reverse Genetics System for a Human Group A Rotavirus. J. Virol..

[31] Falkenhagen A., Patzina-Mehling C., Ruckner A., Vahlenkamp T.W., Johne R. (2019). Generation of simian rotavirus reassortants with diverse VP4 genes using reverse genetics. J. Gen. Virol..

[32] Potgieter A.C., Page N.A., Liebenberg J., Wright I.M., Landt O., van Dijk A.A. (2009). Improved strategies for sequence-independent amplification and sequencing of viral double-stranded RNA genomes. J. Gen. Virol..

[33] Jere K.C., Mlera L., O’Neill H.G., Potgieter A.C., Page N.A., Seheri M.L., van Dijk A.A. (2011). Whole genome analyses of African G2, G8, G9, and G12 rotavirus strains using sequence-independent amplification and 454(R) pyrosequencing. J. Med. Virol..

[34] Strydom A., Motanyane L., Nyaga M.M., Joao E.D., Cuamba A., Mandomando I., Cassocera M., de Deus N., O’Neill H. (2019). Whole-genome characterization of G12 rotavirus strains detected in Mozambique reveals a co-infection with a GXP[14] strain of possible animal origin. J. Gen. Virol..

[35] Strydom A., Joao E.D., Motanyane L., Nyaga M.M., Christiaan Potgieter A., Cuamba A., Mandomando I., Cassocera M., de Deus N., O’Neill H.G. (2019). Whole genome analyses of DS-1-like Rotavirus A strains detected in children with acute diarrhoea in southern Mozambique suggest several reassortment events. Infect. Genet. Evol..

[36] Joao E.D., Strydom A., O’Neill H.G., Cuamba A., Cassocera M., Acacio S., Mandomando I., Motanyane L., Page N., de Deus N. (2018). Rotavirus A strains obtained from children with acute gastroenteritis in Mozambique, 2012–2013: G and P genotypes and phylogenetic analysis of VP7 and partial VP4 genes. Arch. Virol..

[37] Aoki S.T., Settembre E.C., Trask S.D., Greenberg H.B., Harrison S.C., Dormitzer P.R. (2009). Structure of rotavirus outer-layer protein VP7 bound with a neutralizing Fab. Science.

[38] Zhou X., Wang Y.H., Ghosh S., Tang W.F., Pang B.B., Liu M.Q., Peng J.S., Zhou D.J., Kobayashi N. (2015). Genomic characterization of G3P[6], G4P[6] and G4P[8] human rotaviruses from Wuhan, China: Evidence for interspecies transmission and reassortment events. Infect. Genet. Evol..

[39] Laird A.R., Ibarra V., Ruiz-Palacios G., Guerrero M.L., Glass R.I., Gentsch J.R. (2003). Unexpected detection of animal VP7 genes among common rotavirus strains isolated from children in Mexico. J. Clin. Microbiol..

[40] Steyer A., Poljsak-Prijatelj M., Barlic-Maganja D., Marin J. (2008). Human, porcine and bovine rotaviruses in Slovenia: Evidence of interspecies transmission and genome reassortment. J. Gen. Virol..

[41] Midthun K., Greenberg H.B., Hoshino Y., Kapikian A.Z., Wyatt R.G., Chanock R.M. (1985). Reassortant rotaviruses as potential live rotavirus vaccine candidates. J. Virol..

[42] Kobayashi N., Kojima K., Taniguchi K., Urasawa T., Urasawa S. (1994). Genotypic diversity of reassortants between simian rotavirus SA11 and human rotaviruses having different antigenic specificities and RNA patterns. Res. Virol..

[43] Nyaga M.M., Tan Y., Seheri M.L., Halpin R.A., Akopov A., Stucker K.M., Fedorova N.B., Shrivastava S., Duncan Steele A., Mwenda J.M. (2018). Whole-genome sequencing and analyses identify high genetic heterogeneity, diversity and endemicity of rotavirus genotype P[6] strains circulating in Africa. Infect. Genet. Evol..

[44] Gombold J.L., Estes M.K., Ramig R.F. (1985). Assignment of simian rotavirus SA11 temperature-sensitive mutant groups B and E to genome segments. Virology.

[45] Greenberg H.B., Flores J., Kalica A.R., Wyatt R.G., Jones R. (1983). Gene coding assignments for growth restriction, neutralization and subgroup specificities of the W and DS-1 strains of human rotavirus. J. Gen. Virol..

[46] Gorrell R.J., Bishop R.F. (1997). Production of reassortant viruses containing human rotavirus VP4 and SA11 VP7 for measuring neutralizing antibody following natural infection. Clin. Diagn. Lab. Immunol..

[47] Jiang X., Liu Y., Tan M. (2017). Histo-blood group antigens as receptors for rotavirus, new understanding on rotavirus epidemiology and vaccine strategy. Emerg. Microbes Infect..

[48] Huang P., Xia M., Tan M., Zhong W., Wei C., Wang L., Morrow A., Jiang X. (2012). Spike protein VP8* of human rotavirus recognizes histo-blood group antigens in a type-specific manner. J. Virol..

[49] Barbe L., le Moullac-Vaidye B., Echasserieau K., Bernardeau K., Carton T., Bovin N., Nordgren J., Svensson L., Ruvoen-Clouet N., le Pendu J. (2018). Histo-blood group antigen-binding specificities of human rotaviruses are associated with gastroenteritis but not with in vitro infection. Sci. Rep..

[50] Hu L., Crawford S.E., Czako R., Cortes-Penfield N.W., Smith D.F., le Pendu J., Estes M.K., Prasad B.V. (2012). Cell attachment protein VP8* of a human rotavirus specifically interacts with A-type histo-blood group antigen. Nature.

[51] Bohm R., Fleming F.E., Maggioni A., Dang V.T., Holloway G., Coulson B.S., von Itzstein M., Haselhorst T. (2015). Revisiting the role of histo-blood group antigens in rotavirus host-cell invasion. Nat. Commun..

[52] Simwaka J.C., Mpabalwani E.M., Seheri M., Peenze I., Monze M., Matapo B., Parashar U.D., Mufunda J., Mphahlele J.M., Tate J.E. (2018). Diversity of rotavirus strains circulating in children under five years of age who presented with acute gastroenteritis before and after rotavirus vaccine introduction, University Teaching Hospital, Lusaka, Zambia, 2008–2015. Vaccine.

[53] Lartey B.L., Damanka S., Dennis F.E., Enweronu-Laryea C.C., Addo-Yobo E., Ansong D., Kwarteng-Owusu S., Sagoe K.W., Mwenda J.M., Diamenu S.K. (2018). Rotavirus strain distribution in Ghana pre- and post- rotavirus vaccine introduction. Vaccine.

[54] Mukaratirwa A., Berejena C., Nziramasanga P., Ticklay I., Gonah A., Nathoo K., Manangazira P., Mangwanya D., Marembo J., Mwenda J.M. (2018). Distribution of rotavirus genotypes associated with acute diarrhoea in Zimbabwean children less than five years old before and after rotavirus vaccine introduction. Vaccine.

[55] Bhandari N., Rongsen-Chandola T., Bavdekar A., John J., Antony K., Taneja S., Goyal N., Kawade A., Kang G., Rathore S.S. (2014). Efficacy of a monovalent human-bovine (116E) rotavirus vaccine in Indian infants: A randomised, double-blind, placebo-controlled trial. Lancet.

[56] Kulkarni P.S., Desai S., Tewari T., Kawade A., Goyal N., Garg B.S., Kumar D., Kanungo S., Kamat V., Kang G. (2017). A randomized Phase III clinical trial to assess the efficacy of a bovine-human reassortant pentavalent rotavirus vaccine in Indian infants. Vaccine.

[57] Bhan M.K., Lew J.F., Sazawal S., Das B.K., Gentsch J.R., Glass R.I. (1993). Protection conferred by neonatal rotavirus infection against subsequent rotavirus diarrhea. J. Infect. Dis..

[58] Zade J.K., Kulkarni P.S., Desai S.A., Sabale R.N., Naik S.P., Dhere R.M. (2014). Bovine rotavirus pentavalent vaccine development in India. Vaccine.

[59] Feng N., Hu L., Ding S., Sanyal M., Zhao B., Sankaran B., Ramani S., McNeal M., Yasukawa L.L., Song Y. (2019). Human VP8* mAbs neutralize rotavirus selectively in human intestinal epithelial cells. J. Clin. Investig..

